# Susceptibility of HBZ and Tax expressing primary HTLV-1+ T cell clones to CTL lysis

**DOI:** 10.1186/1742-4690-8-S1-A105

**Published:** 2011-06-06

**Authors:** Aileen G Rowan, Becca Asquith, Graham P Taylor, Charles R M Bangham

**Affiliations:** 1 Department of Immunology, Imperial College London, W2 1PG, UK; 2 Section of Infectious Diseases, Imperial College London, W2 1PG, UK

## 

Host cytotoxic lymphocyte (CTL) responses play an essential role in limiting clonal expansion of infected CD4+ T cells in HTLV-1+ individuals. We have reported that individuals who possess HLA class 1 alleles that strongly bind peptides from HTLV-1 basic leucine zipper protein (HBZ) have a lower proviral load and are less likely to develop HAM/TSP. In general, peptides from HBZ bind weakly to MHCI, and we detected low frequencies of HBZ-specific CD8+ T cells in ≈25% of infected individuals. Despite its poor immunogenicity, high avidity CTL lines can be generated in vitro which efficiently lyse HBZ-presenting cells [1].

HBZ mRNA, encoded on the antisense strand of the viral genome, can induce proliferation of T cells, and once translated it can inhibit expression of Tax. HBZ mRNA is expressed in vivo in the majority of infected individuals, however, HBZ protein expression levels are extremely low in PBMCs ex vivo. To investigate the relationship between Tax and HBZ expression in naturally infected CD4+ T cells at the single cell level, we cloned T cells from individuals with HAM/TSP. We detected HBZ mRNA in all clones tested. In contrast, a subset of infected clones stably expressed Tax protein. Thus, individual clones may have a unique viral gene expression profile, with differing susceptibility to lysis by HTLV-1 specific CTL. We are testing the hypothesis that targeting of Tax– clones by HBZ-specific CTL eliminates clones which otherwise escape lysis by abundant Tax-specific CTL.
